# Polymorphism of Transcription Factor-7-Like 2 (TCF7L2) Gene and New-Onset Diabetes after Liver Transplantation

**Published:** 2015-02-01

**Authors:** Z. Musavi, N. Azarpira, M. H. Sangtarash, M. Kordi, K. Kazemi, B. Geramizadeh, S. A. Malek-Hosseini

**Affiliations:** 1 Transplant Research Center, Shiraz University of Medical Sciences, Shiraz, Iran; 2 *Department of Genetic, Sistan and Baluchestan University of Sciences, Zahedan, Iran*

**Keywords:** Transcription factor 7-like 2 protein, Diabetes mellitus, Polymorphism, genetic, Liver transplantation, Transcription factors

## Abstract

Background: New-onset diabetes after transplantation (NODAT) is a serious complication in transplant recipients. Transcription factor-7-like 2 (TCF7L2) is a Wnt signaling-associated transcription factor that plays an important role in β-cell proliferation and insulin secretion. The association between TCF7L2 SNP rs7903146 and NODAT was documented in renal transplant patients.

Objective: To determine the association between TCF7L2 rs7903146 variants and the risk of NODAT after liver transplantation.

Methods: This study was conducted on 140 liver transplant recipients who had received tacrolimus-based immunosuppressive drugs. The patients were divided into NODAT (n=70) and non-NODAT (n=70) groups and were genotyped using PCR-RFLP. In addition, 100 normal subjects were considered as the comparison group.

Results: There was a significant difference (p<0.05) between the two study groups regarding donor and recipient age, recipient body mass index, and recipient fasting plasma glucose before the transplantation. No significant relationship was observed between *TCF7L2 *rs7903146 genotypes and development of NODAT. No significant difference was also found between the two groups in terms of the tacrolimus and mycophenolate mofetil daily dosage as well as tacrolimus blood level. However, the prednisolone daily dosage was significantly (p=0.01) higher in the NODAT group compared to those without NODAT. The majority of the patients in the NODAT group also had an episode of acute rejection. Furthermore, a significant difference was found between the transplant recipients and the comparison subjects regarding T allele (p<0.001, OR=1.96) and TT genotype (p<0.001, OR=3.47) frequencies.

Conclusion: No correlation was found between *TCF7L2 *genotypes and development of NODAT. Acute rejection and prednisolone pulse therapy predisposed the susceptible patients to NODAT.

## INTRODUCTION

New-onset diabetes after transplantation (NODAT) is a frequent complication in recipients of organ or cellular transplantation [1, 2]. The development of NODAT has been reported to be associated with increased graft loss and decreased graft and patient survival [1]. A large meta-analysis indicated that the prevalence of NODAT ranged from 2% to 50% in patients undergoing solid organ transplantation at 1-year post-transplantation [1, 3].

The prevalence of NODAT has been reported to be 4%–25% in renal transplant recipients, 2.5%–25% in liver transplant recipients, 4%–40% in heart transplant recipients, and 30%–35% in lung transplant recipients [3, 4, 5].

The pathophysiology of NODAT closely resembles that of type 2 diabetes mellitus (T2DM), which is characterized by a combination of insulin resistance and insulin hyposecretion. NODAT is a multifactorial disease caused by both genetic and non-genetic factors [4].

The risk factors of NODAT include age above 40, obesity, high body mass index (BMI), ethnicity (Black and Hispanic), family history of T2DM, and cytomegalovirus or hepatitis C virus (HCV) infection [4, 5]. 

Immunosuppressive drugs contribute to the development of NODAT through different mechanisms including insulin resistance (by corticosteroids), and decrease in insulin secretion (mainly by tacrolimus) [4, 5]. The Genome-Wide Association (GWA) studies and meta-analyses have identified many genetic variants associated with the risk of T2DM [6-9]. 

Transcription factor 7-like 2 (*TCF7L2*) gene located on chromosome 10q25.3, has been considered as one of the major diabetes susceptibility genes [10]. Two large meta-analyses revealed strong associations between TCF7L2 SNP rs7903146 and development of T2DM [11, 12].

TCF7L2 is a Wnt signaling-associated transcription factor, which is important in pancreatic islet development [13] and induces the expression of genes encoding the glucagon-like peptide 1 as well as those that are involved in processing and exocytosis of insulin granules [14, 15].

We conducted this study to determine the association between variations in *TCF7L2* and the development of NODAT among Iranian liver allograft recipients.

## PATIENTS AND METHODS

This study was conducted on 400 liver transplant recipients transplanted during 2011 to 2013. The Ethics Committee of Shiraz University of Medical Sciences approved the protocol. Furthermore, informed written consents were taken from all study participants. The patients were eligible to participate in the study if they had no previous diagnosis of diabetes, had a pre-transplantation fasting plasma glucose (FPG) level of <5.5 mmol/L (99 mg/dL), and were followed for at least 10 months. Those with history of diabetes mellitus hyperthyroidism, hypothyroidism, Cushing syndrome, or pheochromocytoma prior to the transplantation were excluded from the study. After considering these screening criteria, a total of 140 liver allograft recipients were enrolled into the study. 

The patients received a treatment protocol consisting of tacrolimus, mycophenolate mofetil, and steroids. Tacrolimus was started at 0.1 mg/kg per day with doses adjusted to keep a trough level between 10 and 12 ng/mL in the first post-transplant month and subsequently between 8 and 10 ng/mL. Additionally, mycophenolate mofetil was given with an initial oral dose of 2.0 g/day in equally divided doses every 12 h. Finally, methylprednisolone 1 g, was given on the day of surgery, and prednisolone, 20 mg tapered down to zero within the first three months. 

According to the American Diabetes Association criteria [16], NODAT was defined as a fasting glucose level of at least 7 mmol/L (126 mg/dL) or a non-fasting glucose level of at least 11.1 mmol/L (200 mg/dL) confirmed on at least two occasions, or consumption of anti-diabetic drugs beyond the first month after transplantation.

The liver transplant recipients were divided into NODAT (n=70) and non-NODAT (n=70) groups. Besides, 100 persons with no past history of glucose intolerance were considered as the control group. 

Genomic DNA was extracted from buffy coats using a Genomic DNA purification DNPTM kit (Cinagene, Iran). In addition, *TCF7L2 *rs7903146 was genotyped through PCR-RFLP method. The primer sequences used were as follows:

**TCF7L2 F:** 5′TTAGAGAGCTAAGCACTTTTTAGGTA3′ and 

**TCF7L2 R:** 5′AGCTTCTCAGT-CACACAGGC3′

PCR was carried out under the following conditions: a denaturation step at 95 °C for 5 min, and 35 cycles at 95 °C for 45 sec, 61 °C for 1 min, and 72°C for 1 min. The PCR products were analyzed by electrophoresis on 4% agarose gels stained with ethidium bromide. 

The PCR products were then digested with RSaI (Biolab, New England) at 37 °C for 16 h and were observed using electrophoresis and ethidium bromide. The PCR product was 171 bp. After digestion, the CT genotype was 171 bp, 146 bp, and 45 bp; the TT genotype was 171 bp; and the CC genotype was 146 bp and 45 bp.

Afterwards, one product from each genotype was sequenced with ABI sequence Genetic Analyzer (Applied Biosystems, Foster City, CA, USA) to confirm the results and decrease the personal as well as the instrumental errors.

Statistical analysis

Quantitative variables were expressed as mean±SD; the categorical variables were presented as frequency (%). The quantitative variables were compared using *Student’s t* test; categorical variables were compared by χ^2^ test. Logistic regression analysis was used to evaluate the risk factors. The variables with statistical significance in the univariate analyses were entered a stepwise multivariate regression analysis. Alerquin software was used to analyze the Hardy-Weinberg equilibrium. All the statistical analyses were performed using the SPSS^®^ for Windows^®^ ver 18 (SPSS Inc, Chicago, IL, USA). A p value <0.05 was considered statistically significant.

## RESULTS

The overall incidence of NODAT was 17.5% (n=70: 95% CI: 13.8% 21.2%) within the first year after liver transplantation. There was a significant difference between NODAT and non-NODAT groups in terms of donor age (p<0.001), patients’ mean age (p<0.001), recipient BMI (p=0.007), frequency of acute rejection episode (p=0.011), and recipients’ pre-transplant fasting plasma glucose level (p=0.043) (Table 1). 

**Table 1:** Demographic characteristics of patients enrolled in the study. Figures are either mean±SD or frequency (%).

Parameter	NODAT (n=70) n (%)	No NODAT (n=70) n (%)	p-value
Recipient Sex			
Male	35 (50)	44 (62)	0.125
Female	35 (50)	26 (37)	
Recipient age	45.3±13.2	32.5±17.1	0.001
Donor sex			
Male	56 (80)	47(67)	0.11
Female	14 (20)	23 (33)	
Donor age	45.3±13.2	27.48±11.99	0.001
Blood group			
A	23 (33)	28 (40)	0.03
B	15 (21)	26 (37)	
AB	7 (10)	3 (4)	
O	25 (36)	13 (19)	
MELD score	45.30±13.24	21.41±5.50	0.817
BMI (kg/m^2^)	45.30±13.24	21.17±4.85	0.007
Acute rejection episode	24 (34)	11 (16)	0.011
Viral infection*	4 (5)	0	0.001
Immunosuppressive drugs			
Tacrolimus dose	3.15±1.38	3.36±1.21	0.307
Mycophenolate mofetil dose	1.91±1.06	3.62±12.98	0.736
Prednisolone dose	11.22±4.10	9.35±3.55	0.005
Blood tacrolimus level	10.32±4.11	12.15±4.71	0.11

* Positive CMV antigenemia that means active infection.

No significant difference was observed between the two groups in terms of other baseline characteristics including donor gender and patient parameters (MELD score, blood group). Nonetheless, active cytomegalovirus infection was more prevalent in the NODAT group. Also, more steroid-treated acute rejection episodes were observed in patients with NODAT (p*=*0.011) (Table 1). Due to missing data, the evaluation of family history was not possible.

The FPG (p<0.001) and total triglyceride levels (p=0.01) were significantly higher in the NODAT group compared to the non-NODAT patients both one and six months of the transplantation (Table 2). However, there was no significant difference between the two groups in terms of the tacrolimus and mycophenolate mofetil daily dosage. Yet, the prednisolone daily dosage was significantly (p=0.01) higher in the NODAT group in comparison to the non-NODAT group. The majority of patients in NODAT group also had an episode of acute rejection and received pulse of methylprednisolone. No significant difference was observed between the two groups with respect to blood tacrolimus levels (Table 1).

**Table 2:** Biochemical parameters before and after liver transplantation. Figures are mean±SD.

Parameter	Time (relative to transplantation time)	NODAT	No NODAT	p value
Fasting plasma glucose (mg/dL)	Before	95±15.97	88.36±21.97	0.043
	1 month after	164.26±64.68	86.78±14.59	0.001
	6 months after	135.57±66.32	88.44±12.63	0.001
Triglycerides (mmol/L)	Before	100.78±57.81	94.27±51.85	0.324
	1 month after	203.78±88.21	167.16±76.71	0.015
	6 months after	166.78±95.93	129.43±64.03	0.014
Total cholesterol (mmol/L)	Before	136.35±47.80	146.32±104.2	0.475
	1 month after	185.67±57.73	163.84±48.52	0.02
	6 months after	184.36±103.13	150.09±50.62	0.002
HDL-cholesterol (mmol/L)	Before	41.73±32.03	46.49±27.22	0.249
	1 month after	44.22±14.16	49.88±22.61	0.17
	6 months after	44.49±12.59	46.90±22.27	0.83
LDL-cholesterol (mmol/L)	Before	76.76±31.55	88.72±51.59	0.19
	1 month after	94.02±36.53	92.44±36.18	0.846
	6 months after	110.75±90.56	92.79±58.07	0.081

The genotype frequency distributions did not show deviation from the Hardy-Weinberg equilibrium, either in normal study subjects or in the groups categorized according to their diabetes status. Allele and genotype frequencies for SNP rs7903146 were similar in all the patients (with and without T2DM) (Table 3). However, a significant difference was found between the transplant recipients and the comparison subjects regarding the frequency of T allele (p<0.001, OR=1.96) and TT genotype (p<0.001, OR=3.47) (Table 4).

**Table 3:** Different genotypes and allele frequencies of TCF7L2 gene polymorphism in liver transplant recipients

Rs7903146 polymorphism	No NODAT (n=70) n (%)	NODAT (n=70) n (%)	OR (95%CI)	p value
Co-dominant				
CC	19 (27)	19 (27)	1.00 (0.44–2.25)	1.00
CT	36 (51)	37 (53)	0.94 (0.46–1.93)	0.86
TT	15 (21)	14 (20)	1.09 (0.45–2.67)	0.83
Dominant model				
CC	19 (27)	19 (27)	1.00 (0.44–2.25)	1.00
CT+TT	51 (72)	51 (73)		
Recessive model				
CC+CT	55 (79)	56 (80)	1.09 (0.45–2.67)	0.83
TT	15 (21)	14 (20)		
Alleles				
C	74	75	0.97 (0.59–1.60)	0.90
T	66	65		

**Table 4:** Distribution of rs7903146 TCF7L2 genotypes and alleles between normal population, NODAT and all liver transplant recipients

rs7903146 polymorphism		T2DM (n=70) n (%)	Normal population (n=100) n (%)	p value*	OR (95% CI)	All liver transplant recipients (n=140) n (%)	p value^†^	OR (95% CI)
Genotype	CC	19 (27)	45 (45)	0.01	0.46 (0.22–0.92)	38 (27.1)	0.004	0.46 (0.26–0.81)
	CT	36 (51)	48 (48)	0.65	1.15 (0.59–2.21)	73 (52.1)	0.52	1.18 (0.68–2.04)
	TT	15 (21)	7 (7)	0.005	3.62 (1.28–10.54)	29 (20.7)	0.003	3.47 (1.37–9.15)
Allele	T	66 (47)	62 (31)	0.002	1.99 (1.24–3.18)	131 (47.0)	0.0005	1.96 (1.31–2.92)
	C	74 (53)	138 (69)			149 (53.0)		

* Comparison between normal population and NODAT;

† Comparison between normal population and all liver transplant recipients

## DISCUSSION

TCF7L2 protein plays an important role in regulation of cell proliferation and differentiation through the Wnt signaling pathway. It is also implicated in development and maturation of the pancreas, including the islets of Langerhans and beta cells [17]. Moreover, it affects insulin secretion through its functional effect on glucagon-like peptide-1 (GLP-1) signaling in beta cells [14, 15, 17, 18]. The variants of *TCF7L2 *gene have been reported to be associated with T2DM in different populations [10-14], with the strongest association being related to the SNP rs7903146 T allele. This SNP is located in a noncoding region with no obvious mutational mechanisms [10, 12].

The results of the current study revealed a significant association between several parameters and NODAT. The recipients’ mean age, BMI, frequency of acute rejection episode, and total steroid dose were significantly higher in the NODAT group compared to non-NODAT patients. These parameters are well known risk factors of NODAT in transplant recipients [1, 4]. However, in this study, no significant relationship was observed between *TCF7L2 *rs7903146 genotype and NODAT. Chakkera and colleagues analyzed the *TCF7L2 *rs7903146 gene variant in a cohort of kidney transplant patients. They found no association between *TCF7L2 *variants and NODAT [19]. 

Moreover, Kurzawski, *et al*, assessed NODAT in Polish renal transplant recipients and divided the recipients into NODAT and non-NODAT. The medication protocol consisted of tacrolimus, mycophenolate mofetil, and steroids [20]. The results showed no significant difference between the study groups in terms of the frequency of rs7903146 SNPs [20].

Ghisdal, *et al* [21], similar to Kang, *et al* [22], reported a significant association between the *CF7L2 *rs7903146 polymorphism and NODAT in Korean renal transplant recipients. Ghisdal and colleagues [21], conducted their study on kidney recipients of predominantly Caucasian origin and their results demonstrated a significant relationship between NODAT and T allele, patient’s age, BMI, tacrolimus use, steroid boluses, and acute rejection episodes. The two aforementioned studies [21, 22], were performed on the patients who had been treated with cyclosporine, tacrolimus, and sirolimus. Ghisdal and colleagues pointed out that consumption of tacrolimus was an independent risk factor of NODAT [21]. 

In the study by Ghisdal and colleagues [21], the frequency of acute rejection episodes and the total steroid dose were increased in the NODAT patients. In the same line, our study confirmed that methylprednisolone dose and acute rejection episodes were significantly higher in the NODAT group.

Some studies have emphasized that higher levels of tacrolimus were associated with an increased risk of NODAT [21]. In the present study, however, tacrolimus blood levels were similar in all the study groups. In the literature review, only one study was conducted on liver transplant recipients. In that study, which was performed by Ling and colleagues in Chinese population, four SNPs (rs290487, rs7903146, rs11196205, and rs12255372) of TCF7L2 gene were assessed in both donors and recipients [23].

The results of that study indicated that the clinical parameters, such as donor fatty liver, recipient age, blood tacrolimus levels, and donor and recipient rs290487 genetic variation, were the potential influencing factors. However, no significant difference was found between NODAT and non-NODAT groups with respect to distribution of SNP rs7903146 [23].

SNP rs7903146 (T allele) has been confirmed to increase the risk of T2DM, especially in the populations of European ancestry [8]. Unlike in Caucasians, the other two studied SNPs (rs11196205, and rs12255372) have been reported to be associated with diabetes in Eastern Asian populations. In liver transplant recipients, however, these SNPs were not associated with increased risk of NODAT [23]. Other genes such as KCNJ11 E23K polymorphism was also considered as NODAT susceptibility gene [24].

In this study, the distribution of *TCF7L2 *rs7903146 variants in the Iranian population was compared to the data obtained from other populations [9, 25-30] (Table 5). Accordingly, the C variant was the most common detrimental allele among the Caucasians. The frequency of C variant was 69%–71% in the Iranian population, which is similar to the reports of Caucasian, Indian, central Asian, and African populations (Table 5). In addition, the frequency of C variant has been reported to be 0.98 in Eastern Asian countries, including China and Japan [5, 25-31].

**Table 5:** Distribution of rs7903146 TCF7L2 genotypes and alleles frequency in different ethnic groups

Population	Allelic frequency	
	C	T
Central Asia [24] Kyrgyz Tajik	0.9 0.83	0.1 0.17
Iceland [25]	0.7	0.3
Chinese (East Asia) [24]	0.98	0.02
Japanese (East Asia) [26]	0.96	0.04
Brazil [28]	0.73	0.27
Denmark [25]	0.74	0.26
Caucasian [26]	0.7	0.3
South Africa [29]	0.64	0.36
India [30]	0.79	0.21
Iran [31]	0.71	0.29
Current Study	0.69	0.31

Finally, the NODAT is a multifactorial disease. Family history of diabetes, obesity, and type of immunosuppressive drugs are considered as important risk factors. Regular blood glucose monitoring is important to find early glucose intolerance and modification in the immunosuppressant regimen as well as lifestyle change in diet, exercise and weight control can prevent the development of NODAT [32].

In conclusion, we found no association between* TCF7L2 *rs7903146 genotypes and development of NODAT. Acute rejection and prednisolone pulse therapy predisposed the susceptible patients to NODAT. Moreover, T allele and TT genotype were significantly higher in transplant recipients compared to the comparison group. This may predispose the patients to end-stage liver disease. However, these findings should be verified in future independent studies.
